# Lyn prevents aberrant inflammatory responses to *Pseudomonas* infection in mammalian systems by repressing a SHIP-1-associated signaling cluster

**DOI:** 10.1038/sigtrans.2016.32

**Published:** 2016-12-16

**Authors:** Rongpeng Li, Lizhu Fang, Qinqin Pu, Ping Lin, Austin Hoggarth, Huang Huang, Xuefeng Li, Guoping Li, Min Wu

**Affiliations:** 1 Department of Biomedical Sciences, University of North Dakota, Grand Forks, North Dakota, USA; 2 Key Laboratory of Biotechnology for Medicinal Plants of Jiangsu Province, Jiangsu Normal University, Xuzhou, Jiangsu 221116, P.R., China; 3 State Key Laboratory of Biotherapy and Cancer Center, West China Hospital, Sichuan University, and Collaborative Innovation Center for Biotherapy, Chengdu, China; 4 Institute of Human Virology, Sun Yat-sen University, Guangzhou, China; 5 Inflammation and Allergic Disease Research Unit, First Affiliated Hospital of Southwest Medical University, Luzhou, China

## Abstract

The pleiotropic Src kinase Lyn has critical roles in host defense in alveolar macrophages against bacterial infection, but the underlying mechanism for Lyn-mediated inflammatory response remains largely elusive. Using mouse *Pseudomonas aeruginosa* infection models, we observed that Lyn^−/−^ mice manifest severe lung injury and enhanced inflammatory responses, compared with wild-type littermates. We demonstrate that Lyn exerts this immune function through interaction with IL-6 receptor and cytoskeletal protein Ezrin via its SH2 and SH3 domains. Depletion of Lyn results in excessive STAT3 activation, and enhanced the Src homology 2-containing inositol-5-phopsphatase 1 (SHIP-1) expression. Deletion of SHIP-1 in Lyn^−/−^ mice (double knockout) promotes mouse survival and reduces inflammatory responses during *P. aeruginosa* infection, revealing the rescue of the deadly infectious phenotype in Lyn deficiency. Mechanistically, loss of SHIP-1 reduces NF-κB-dependent cytokine production and dampens MAP kinase activation through a TLR4-independent PI3K/Akt pathway. These findings reveal Lyn as a regulator for host immune response against *P. aeruginosa* infection through SHIP-1 and IL-6/STAT3 signaling pathway in alveolar macrophages.

## Introduction

*Pseudomonas aeruginosa* is an opportunistic bacterium causing acute and chronic infection in immunocompromised people,^1^ such as patients with cystic fibrosis, chronic obstructive pulmonary disease, severe burns and cancer. *P. aeruginosa* infection is the major cause of the morbidity and mortality of these diseases.^2^ Innate immune responses, including inflammatory cytokine production, immune cell recruitment and phagocytic clearance by neutrophils and macrophages, have critical roles in host defense during the early stages of infection and profoundly influence the generation of the adaptive immune responses and disease outcomes.^3–5^ The pattern recognition receptors are important factors for recognizing pathogen-associated molecular patterns, such as lipopolysaccharide (LPS), and initiating innate (frontline) immunity to battle against pathogens, and subsequently transmitting signals for antigen-specific adaptive immune responses to provide a second layer of protection.^6^

Lyn, a member of the Src family of nonreceptor tyrosine kinases, is an important regulator of immune homeostasis and pattern recognition receptor-induced responses.^7^ Previous studies have determined that Lyn is involved in B-cell responses,^8^ proliferation and degranulation of mast cells,^9^ integrin signaling in neutrophils,^10^ M2 macrophage polarization,^11^ dendritic cell function, and NK cell activation.^12^ Lyn is activated upon ligand binding to a wide variety of cell surface receptors that are essential for promoting or limiting immune responses.^13,14^ A recent study showed that Lyn can activate Abl2/Arg to facilitate IgG-mediated phagocytosis and *Leishmania* infection.^15^ Another work indicated that Lyn is also relevant to microbiota-dependent intestinal inflammation and susceptibility to enteric pathogens.^16^

Lyn has roles during pulmonary infections. Previous reports from us and others showed that Lyn is located on the inner leaflet of the plasma membrane and in the proximity of lipid rafts, and can thus be translocated into the activated membrane domains to transmit cellular signals for either facilitating phagocytosis or regulating inflammatory responses.^17–19^ Upon *Klebsiella pneumoniae* infection, Lyn negatively regulates inflammatory responses via the p38/NF-κB signaling pathway to maintain a balanced inflammation.^20^ Lyn is also critical for *P. aeruginosa* internalization into lung cells.^21^ Lyn is activated to be associated with lipid rafts and TLR2, playing an important role in the initial stages of *P. aeruginosa* infection in both alveolar macrophages (AMs) and lung epithelial cells. Our more recent study showed that Lyn facilitates the delivery of both *K. pneumoniae* and *P. aeruginosa* to lysosomes for eradication through TLR2-initiated autophagy-related phagocytosis.^22^ However, the molecular mechanism in Lyn-mediated signaling pathway during *P. aeruginosa* infection is still largely unclear.

Using murine gene knockout models, here we set up to elucidate the molecular mechanism by which Lyn regulates host defense during *P. aeruginosa*-induced acute pneumonia. We demonstrate that Lyn directly binds to interleukin-6 receptor (IL-6R) to regulate IL-6/STAT3 signaling pathway in AMs. Furthermore, we found that Lyn regulates nuclear factor-κB (NF-κB) and MAP kinase (MAPK) activation by controlling the activation of cytosolic phosphatase SH2-containing inositol-5′-phosphatase 1 (SHIP-1). During *P. aeruginosa* infection, SHIP-1 positively regulates NF-κB signaling pathway, but negatively regulates MAPK signaling pathway through Akt activation. These results provide new insight into the function of Lyn in bacterial infection.

## Materials and methods

### Mice

C57BL/6J mice (6–8 weeks) were obtained from the Jackson Laboratory (Bar Harbor, ME, USA). Lyn^−/−^ and Ship-1^−/−^ mice that are constructed based on C57BL/6J mice were kindly provided by Dr S Li (University of Massachusetts School of Medicine)^23^ and Dr C Baran (Ohio State University), respectively.^24^ Lyn^−/−^/Ship-1^−/−^ (short as L/S^−/−^) mice were generated by cross-breeding Lyn^−/−^ mice with Ship-1^−/−^ mice in our facility using a standard protocol (no abnormal phenotypes noticed). Mice were genotyped by PCR and/or western blotting to confirm the gene deletion before use. Animals were kept in a specific pathogen-free facility of University of North Dakota.^25^ All animal studies were approved by the University of North Dakota Institutional Animal Care and Use Committee (IACUC) and performed in accordance with the animal care and institutional guidelines (IACUC approval #1204-4). The animal experimental procedures including treatment, care and end point choice were followed the Animal Research: Reporting *In Vivo* Experiment guidelines.^25^

### Primary cells and cell lines

Mice were killed, and the thoracic cavity and trachea were dissected. A small incision was made in the trachea via 1-ml syringe with an angiocath (BD Biosciences, Franklin Lakes, NJ, USA), and recovered to a sterile tube. The lungs were lavaged three times with 1 ml of phosphate-buffered saline containing 1% fetal bovine serum (Life Technologies, Grand Island, NY, USA). The retained bronchoalveolar lavage fluid (BALF) was centrifuged at 600 *g* for 5 min at 4 °C. A small smear was made from the BALF and stained with HEMA-3 (ThermoFisher Scientific, Waltham, MA, USA) for cell differential counting according to the manufacture’s instruction.^26^ The cell pellets were resuspended in RPMI 1640 medium (Life Technologies) supplemented with 10% fetal bovine serum and incubated on culture plate for 1 h at 37 °C/5% CO_2_ incubator to allow attachment of macrophages. Non-adherent cells were removed by washing with normal saline. Murine MH-S AM cells were obtained from American Type Culture Collection (Manassas, VA, USA) and cultured following the manufacturer’s instructions.^25^

### Bacteria preparation and infection experiments

The *P. aeruginosa* wild-type (WT) strain, PAO1, was kindly provided by Dr S Lory (Harvard University).^27^ PAK was obtained from Dr G Pier (Harvard University).^28^ Bacteria were grown for about 16 h in lysogeny broth at 37 °C with 220 r.p.m. shaking. The bacteria were pelleted by centrifugation at 5000 *g*. Various mammalian cells were changed to antibiotic-free medium and infected by bacteria in an multiplicity of infection (MOI) of 20:1 bacteria–cell ratio. Mice were anesthetized with 45 mg kg^−1^ ketamine and intranasally instilled 1×10^7^ clonal-forming units (CFU) of PAO1 or 5×10^6^ CFU of PAK in 50 μl phosphate-buffered saline (10 mice per group for survival rates test and three mice per group for other assays), respectively.^17^ Mice were monitored for symptoms and killed when they were moribund.

### RNA isolation and quantitative reverse transcription-PCR

RNA was isolated from indicated MH-S cells. A 50-ng DNA-free RNA was used for the first strand of cDNA synthesis using a SuperScript III first-strand synthesis system (Life Technologies). Quantitative reverse transcription-PCR was performed using the iTag Universal SYBR Green Supermix (Bio-Rad, Hercules, CA, USA) and gene-specific primers (Supplementary Table 1, synthesized in Integrated DNA Technologies, Coralville, IA, USA), in a CFX Connect Real-time PCR Detection System (Bio-Rad). Relative transcript levels were first normalized C_T_ values to GAPDH, and then normalized to the indicated control (2^−ΔΔC^^T^).^25^

### Transfection of siRNA, plasmids, activators and inhibitors

Lyn (SC-35828), Ship-1 (SC-36491), Ezrin (SC-35350) and siNC (SC-37007) short interfering RNAs (siRNAs) were obtained from Santa Cruz Biotechnology (Santa Cruz, CA, USA). MH-S cells were transfected with siRNA (5 pM), p-NF-κB-luc (100 ng) plasmid^29^ using LipofectAmine 2000 (Life Technologies) for 24 h following the manufacture’s instruction. Two hours before PAO1 infection as indicated, MH-S cells were treated with 20 μm STAT3 inhibitor VI (Merck Millipore, Bellerica, MA, USA), NF-κB inhibitor Sulfasalazine (R&D Systems, Minneapolis, MN, USA) and Akt inhibitor VI (Santa Cruz Biotechnology), respectively.

### Bacterial burden assay

AMs from BALF and ground lung, spleen, liver and kidney tissues were homogenized with phosphate-buffered saline and spread on lysogeny broth dishes to enumerate bacterial numbers. The dishes were cultured in a 37°C incubator overnight, and colonies were counted. Triplicates were done for each sample and control.^25^

### Dihydrodichlorofluorescein diacetate assay

Dihydrodichlorofluorescein diacetate dye (Life Technologies) does not normally fluoresce but emits green fluorescence upon reaction with superoxide inside cells. AMs were treated as above and an equal amount of dye was added. After 10-min incubation, fluorescence was measured using a fluorometer, using a 485-nm excitation and 528-nm emission filter.^30^

### 3-(4,5-Dimethylthiazol-2-yl)-2,5-dimethyltetrazolium bromide assay

This assay measures the color change of 3-(4,5-dimethylthiazol-2-yl)-2,5-dimethyltetrazolium bromide (Sigma-Aldrich, St Louis, MO, USA) upon reduction by enzymes to assess the viability of cells. Cells were treated as above, and equal amount of dye was added. After one hour incubation, reaction was terminated by stop solution and left at room temperature overnight for complete dissolution of formazan and absorbance at 560 nm was recorded using a multiscan plate reader to quantify the concentration of superoxide anion.^29^

### Histological analysis

Lung tissues of three independent mice were fixed in 10% formalin (Sigma-Aldrich) for 24 h and then embedded in paraffin using a routine histologic procedure. Five-micrometer sections were cut, stained by standard hematoxylin and eosin and examined for differences in morphology post infection.^25^

### Inflammatory cytokine profiling

Cytokine concentrations of tumor necrosis factor-α (TNF-α), IL-6, IL-1β, MIP-2 and IL-4 were measured by enzyme-linked immunosorbent assay kits stained from eBioscience Co. (San Diego, CA, USA), in samples of BALF collected at the indicated times after infection. BALFs were collected and 100-μl aliquots of samples were added to the coated microtiter wells. The cytokine concentrations were determined with corresponding detection horseradish peroxidase-conjugated antibodies (Abs). The values were read at 450 nm.^25^

### Immunoblotting

Mouse monoclonal Abs against GAPDH (SC-47724), STAT3 (SC-8019), p-STAT3 (SC-8059), Ezrin (SC-58758), IL-6R (SC-374259), GST (SC-374171), SHIP-1 (SC-271426), NF-κB p50 (SC-166588), p-NF-κB p50 (SC-271908), p-p38 (SC-166182), p-ERK (SC-7383), TLR4 (SC-293072) and p-Akt (SC-293125), polyclonal Abs against rabbit polyclonal Abs against Lyn (SC-28790), and goat polyclonal Abs against IL-6 (SC-1265) were obtained from Santa Cruz Biotechnology. The samples derived from cells and lung homogenates were lysed in RIPA buffer, separated by electrophoresis on 12% SDS-polyacrylamide gel electrophoresis gels and transferred to nitrocellulose transfer membranes (GE Amersham Biosciences, Pittsburgh, PA, USA). Proteins were detected by western blotting using primary Abs at a concentration of 1/200 (Santa Cruz Biotechnology) and were incubated overnight. Labeling of the first Abs was detected using relevant secondary Abs conjugated to horseradish peroxidase (Santa Cruz Biotechnology),^31^ detected using ECL regents (Santa Cruz Biotechnology). Phosphorylated and total protein levels were determined and quantified by three independent successive immunoblotting membranes.

### Immunoprecipitation

To obtain whole-cell lysates, indicated AMs and MH-S cells were homogenized in lysis buffer containing phosphatase inhibitor (1:10 000) and protease inhibitors (1:50, ThermoFisher Scientific). Then, total cell lysate were mixed with indicated immunoprecipitation antibody, which were coupled to agarose beads (A/G, 50:50, ThermoFisher Scientific). Immunoprecipitates were separated by SDS-polyacrylamide gel electrophoresis and transferred to nitrocellulose transfer membranes. Proteins were detected using detective Abs and were incubated overnight. Labeling of the first Abs was detected using relevant secondary Abs conjugated to horseradish peroxidase and detected using ECL regents.^25^

### GST-Lyn pull-down assay

GST-Lyn constructs with different functional domains were originally obtained from Dr O Miura (Tokyo Medical and Dental University, Tokyo, Japan) and transformed into *Escherichia coli* BL21 DE3 strain. The GST-Lyn fragments were extracted using immobilized glutathione columns following the manufacturer’s instructions.^22^ Equal whole-cell lysates were incubated with GST-Lyn peptide for protein interactions. The pull-down products were analyzed by immunoblotting with indicated Abs.

### Sucrose density gradients

Triton-soluble and Triton-insoluble cell fractions were prepared using the previously described methods.^32^ In brief, MH-S cells were treated with PAO1 (MOI=20, 30 min) and then lysed in TN1 buffer containing 0.5% Triton X-100. One mililiter the lysate was mixed with 1 ml of 85% sucrose and loaded at the bottom of a Beckman centrifuge tube (Beckman Coulter, Fullerton, CA, USA), and overlaid with 7 ml of 30% sucrose followed by 3.5 ml of 5% sucrose. The gradients were centrifuged for 17 h at 38 000 r.p.m. at 4 °C in a Beckman SW40Ti rotor. Nine fractions (~1.4 ml each) were collected from the top of the gradient and were used for analysis by immunoblotting.

### Immunostaining

Lyn-silenced and control MH-S cells were infected with PAO1 (MPI=20) for 0, 30 and 60 min. Cells were individually incubated with primary anti-SHIP-1 Ab, and then the second fluorescein isothiocyanate-conjugated Abs as described.^25^ Localization of SHIP-1 was observed under an LSM 510 Meta Confocal Microscope (Zeiss, Jena, Thuringia, Germany).

### Statistical analysis

Most experiments were conducted in triplicate. Differences between two groups were compared by one-way analysis of variance (Tukey’s *post hoc*) using GraphPad Prism 5 software (GraphPad Software, San Diego, CA, USA), whereas mice survival rates were calculated using Kaplan–Meier curve.^21^

## Results

### Lyn^−/−^ mice show increased inflammatory responses and severe lung injury following PAO1 infection

**Figure 1:**
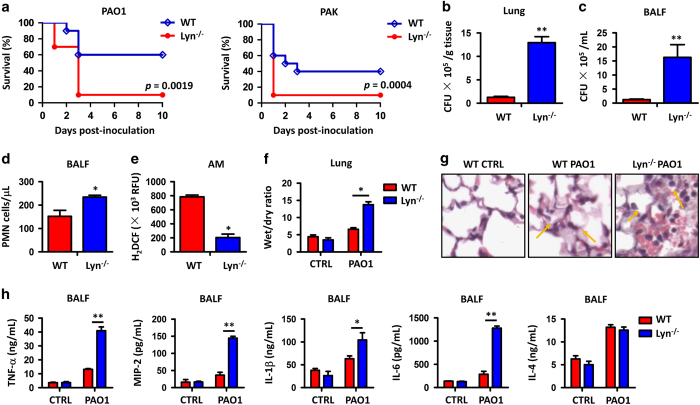
Lyn^−/−^ mice exhibit increased mortality, bacterial burdens, PMNs and intense inflammatory response in PAO1 infection. (**a**) Lyn^−/−^ and WT mice were challenged intranasally with 1×10^7^ CFU PAO1 per mouse (or 5×10^6^ CFU PAK per mouse, 10 mice per group) and then observed to 10 days. Kaplan–Meier survival curves were obtained (*P*=0.0019 in PAO1 infection and *P*=0.0004 in PAK infection). (**b**) At 24 h post PAO1 infection, bacterial burdens assessed using lung homogenates. (**c**) Bacterial loads in BALF of infected mice. (**d**) PMN percentages versus total nuclear cells in BALF measured by HEMA-3 staining. (**e**) Superoxide production in AMs detected by an dihydrodichlorofluorescein diacetate assay. (**f**) Lung edema measured by wet/dry ratio. (**g**) Lung injury and inflammation assessed by histology. Lungs were embedded in formalin and sections were analyzed by hematoxylin and eosin staining (arrows showing typical tissue injury and inflammatory influx). (**h**) Inflammatory cytokines in BALF assessed by enzyme-linked immunosorbent assay. Data (mean±s.e.m.) are representative of three independent experiments (one-way analysis of variance with Tukey’s *post hoc*; *P⩽0.05; **P⩽0.01).

To investigate the physiological relevance of Lyn in *P. aeruginosa* infection, we intranasally instilled laboratory strain PAO1 at 1×10^7^ CFU for each Lyn^−/−^ mouse (Supplementary Figure 1A) as well as WT control mouse (with otherwise similar genetic backgrounds) to establish an acute pneumonia model and compared the survival rates of these two groups of mice (10 mice per group). Figure 1a showed that Lyn^−/−^ mice had a significantly increased mortality compared with WT mice: after 3 days post PAO1 infection, only one Lyn^−/−^ mouse survived, whereas >50% of WT mice remained alive. This result is represented by Kaplan–Meier survival curves (*P*=0.0019, log-rank test). We also examined the survival rate of mice infected by another *P. aeruginosa* strain PAK, which is reported to be more toxic to rodents than PAO1. Similar to PAO1 strain, Lyn^−/−^ mice succumbed to PAK (5×10^6^ CFU per mouse, 10 mice per group) infection compared with WT mice (Figure 1a, *P*=0.0004, log-rank test). These data suggest that Lyn is physiologically relevant to *P. aeruginosa* infection in acute pneumonia models.

To further analyze the cause of infection lethality in Lyn^−/−^ mice, we examined bacterial burdens in the lung, liver, spleen, kidney and BALF from 24 h post PAO1-infected mice. Bacterial CFU increased significantly in the organs of Lyn^−/−^ mice compared with the same organs of WT mice (Figures 1b and c; Supplementary Figures 1B–D), demonstrating severe lung injury and pneumonia associated with Lyn deficiency. Increased recruitment of polymorphonuclear neutrophils (PMNs) into the lung, which is assumed for bacterial clearance, also contributes to severe lung injury and systemic bacterial infection.^33^ To test this idea, we quantified PMN infiltration and observed increased PMNs in the BALF of Lyn^−/−^ mice compared with WT mice (Figure 1d). Phagocyte-released reactive oxygen species (ROS) are crucial for host defense against pathogenic infection by either augmenting antibiotic activity or directly participating in bacterial killing in lysosomes. As determined by dihydrodichlorofluorescein diacetate assay, AMs of Lyn^−/−^ mice showed a significant decrease in oxidative stress at 24 h post infection compared with WT mice (Figure 1e). To assess the extent of acute lung injury, we measured wet/dry ratio of mouse lungs, and found an approximately threefold increase in wet/dry ratio of Lyn^−/−^ mouse lungs versus WT mouse lungs (Figure 1f). In addition, lung histology was examined 24 h post PAO1 infection as a direct indicator of lung injury. Although both Lyn^−/−^ and WT mice showed signs of pneumonia, histological alterations were more severe in the lungs of Lyn^−/−^ mice (Figure 1g). The arrows showed serious inflammatory response due to increased PMN penetration. Finally, we measured cytokine concentrations in BALF 24 h post PAO1 infection to gauge the inflammatory response. The pro-inflammatory cytokines, including TNF-α, MIP-2, IL-1β and IL-6 levels, were significantly elevated in BALF from Lyn^−/−^ mice compared to WT mice, but anti-inflammatory cytokine IL-4 was not altered (Figure 1h), indicating that Lyn preferentially impacts pro-inflammatory cytokine production. Collectively, these data suggest that the severe lung injury in Lyn^−/−^ mice is caused by excessive ROS accumulation and overly active inflammatory response.

### Lyn regulates IL-6/STAT3 signaling pathway by binding with IL-6R and Ezrin

**Figure 2:**
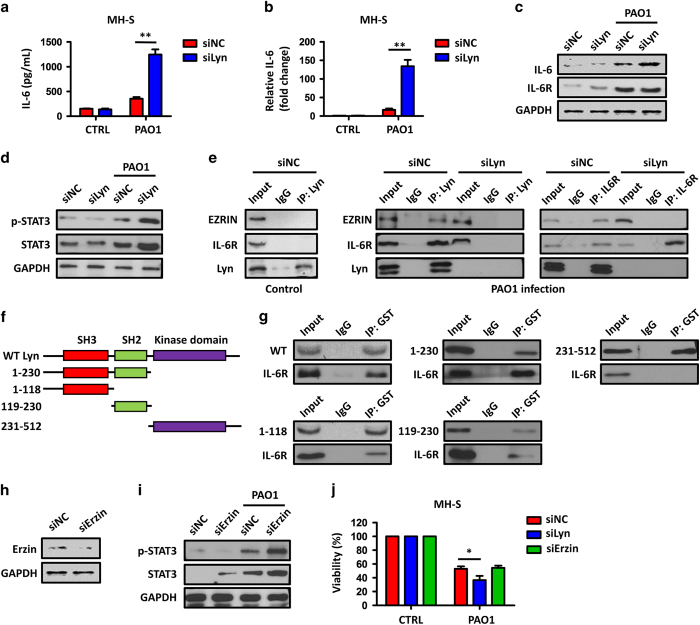
Lyn regulates the IL-6/STAT3 signaling pathway by binding to IL-6R and Ezrin. (**a**) MH-S cells were transfected with control siRNA and Lyn siRNA at 5 pM for 48 h, respectively. The cells were then infected with PAO1 at an MOI of 20:1 for 30 min. IL-6 levels in supernatant were detected by enzyme-linked immunosorbent assay. (**b**) Expression of IL-6 detected by quantitative reverse transcription-PCR. (**c**) Expression of IL-6 and IL-6R was detected by immunoblotting. (**d**) Expression and phosphorylation of STAT3 were measured by immunoblotting. (**e**) Association of Lyn, IL-6R and Ezrin in Lyn-silenced and control MH-S cells was determined by immunoblotting with the indicated Abs for immunoprecipitation and detection in control cells and 30 min post PAO1-infected cells. (**f**) GST-tagged Lyn peptide fragments were used to study *in vitro* association of Lyn with IL-6R. (**g**) MH-S cells infected with PAO1 at an MOI of 20:1 for 30 min and lysed for pull-down assay. GST-Lyn 1–230 (SH2 and SH3). (**h**, **i**) MH-S cells were transfected with control siRNA and Ezrin siRNA at 5 pM for 48 h, respectively (**h**). Ezrin-silenced and control MH-S cells infected with PAO1 at an MOI of 20:1 for 30 min. Expression and phosphorylation of STAT3 measured by immunoblotting (**i**). (**j**) Cell viability of the indicated MH-S cells determined by 3-(4,5-dimethylthiazol-2-yl)-2,5-dimethyltetrazolium bromide assay. Data (mean±s.e.m.) are representative of three independent experiments (one-way analysis of variance with Tukey’s *post hoc*; *P⩽0.05; **P<0.01).

In Figure 1h, PAO1 infection significantly altered inflammatory cytokine IL-6 expression in BALF of Lyn^−/−^ mice, compared with that of WT mice. Considering that the mortality of PAO1-infected mice is highly AM-dependent,^34^ and AM is one of the primary cell population in BALF,^35^ we assumed a critical role of IL-6 regulation by Lyn in these cells during bacterial infection. To confirm this, we transfected murine lung macrophages (MH-S cells) with a siRNA against Lyn to inhibit Lyn expression, as well as with siNC (negative control siRNA) as a negative control (Supplementary Figure S1E). Thirty minutes post PAO1 infection, compared with negative controls, IL-6 production was significantly increased in Lyn-silenced MH-S cells, which was detected by enzyme-linked immunosorbent assay, quantitative PCR and immunoblotting, respectively (Figures 2a–c; Supplementary Figure S2A). However, IL-6R was not significant changed (Figure 2c; Supplementary Figure S2A). As IL-6 activates transcription factor STAT3 to initiate the expression of other inflammatory factors,^36^ we studied whether Lyn regulates STAT3 in AMs during bacterial infection. Immunoblotting showed that after PAO1 infection, the levels of both total and phosphorylated STAT3 were increased in Lyn-silenced MH-S cells, indicating that Lyn regulates the IL-6/STAT3 signaling pathway (Figure 2d; Supplementary Figure S2B).

As Lyn is localized on the cell membrane,^37^ we proposed that Lyn may function via the IL-6/STAT3 signaling pathway by protein–protein interactions with IL-6R (or other cell membrane proteins). To test this potential association mechanism, we lysed control and Lyn-silenced MH-S cells and performed immunoprecipitation assays with anti-Lyn and anti-IL-6R Abs. Although it was not found in lysates from control MH-S cells (without PAO1 infection), a stable interaction between Lyn and IL-6R was detected in siNC-treated MH-S cells after PAO1 infection, but was not found in Lyn-silenced MH-S cells (Figure 2e). Considering Lyn is activated upon ligand binding to a wide variety of cell surface receptors that are essential for immune responses,^13,14^ such as cytoskeletal proteins CDC42 and actin, we presumed that these cell surface factors in AMs may also have an impact on IL-6R/Lyn interaction during PAO1 infection. The co-immunoprecipitation data showed that most results were negative (data not shown), but unexpectedly, Ezrin, a cytosol migration regulator,^38^ was detected in anti-Lyn Abs pulled down products in PAO1-infected WT MH-S cells, but was not found in anti-IL-6R Abs pulled down products in Lyn-silenced MH-S cells or control cells (Figure 2e), suggesting that an Ezrin–Lyn–IL-6R complex is formed in regulation of inflammatory responses against PAO1, and Lyn may bind to both IL-6R and Ezrin. We then tried to determine which domain(s) of Lyn is/are critical for the interaction with IL-6R. Purified Lyn-GST peptides with distinct functional domains (see schematic, Figure 2f) were prepared as described previously.^20,22^ The Lyn-GST fragments were coated on immobilized glutathione agarose beads to pull-down interacting partners from PAO1-infected MH-S cell lysates by anti-GST Abs, and probed using IL-6R Abs. The presence of both Src homolog 2 (SH2) and SH3 domains of Lyn was responsible for interacting with IL-6R upon PAO1 infection in MH-S cells, whereas the kinase domain was not (Figure 2g).

We then asked whether Lyn regulates STAT3 activation through the IL-6R–Lyn–Ezrin complex. Considering IL-6R is critical for IL-6 recognition and binding, here we only treated MH-S cells with siRNAs against Ezrin, to elucidate the inhibitory role of IL-6R–Lyn–Ezrin complex (Figure 2h). Immunoblotting showed that after PAO1 infection, the levels of total and phosphorylated STAT3 were increased in the Ezrin-silenced MH-S cells (Figure 2i; Supplementary Figure S2C), indicating that Lyn indeed regulates the STAT3 activation by binding Ezrin. In addition, 2 h post PAO1 infection, 3-(4,5-dimethylthiazol-2-yl)-2,5-dimethyltetrazolium bromide assay showed a significant decrease in the survivals of Lyn- and Ezrin-silenced MH-S cells, respectively (Figure 2j), indicating that the IL-6R–Lyn–Ezrin complex axis negatively regulates *P. aeruginosa*-induced host inflammatory responses.

### Lyn deficiency results in enhanced PAO1-induced SHIP-1 expression and membrane translocation

**Figure 3:**
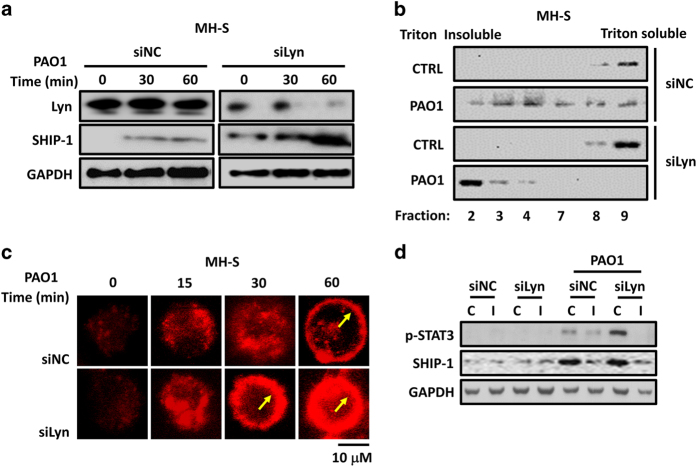
Lyn deficiency leads to enhanced PAO1-induced SHIP-1 expression and membrane translocation. (**a**) MH-S cells were transfected with control siRNA and Lyn siRNA at 5 pM for 48 h, respectively. The cells were infected with PAO1 at an MOI of 20:1 for 0, 30 and 60 min, and expression of SHIP-1 was detected by immunoblotting. (**b**) These cells were infected with PAO1 at an MOI of 20:1 for 30 min, and then lysed for sucrose density gradients (5–30%) assay. Nine fractions were collected from the top of the gradient, and SHIP-1 in Triton X-100-soluble (fractions 2–4) and Triton-insoluble (fractions 7–9) fractions detected by immunoblotting. (**c**) These cells were infected with PAO1 at an MOI of 20:1 for 0, 15, 30 and 60 min. Confocal laser scanning microscopy showed the translocation of SHIP-1 in indicated MH-S cells using immune staining (arrows showing the membrane translocation). Scale bar, 10 μm. (**d**) Cells were treated with STAT3 inhibitor VI (20 μm) before and during PAO1 infection (MOI=20:1, 30 min). Expression of SHIP-1 and phosphorylation of STAT3 were measured by immunoblotting. C, control; I, inhibitor. Data are representative of three independent experiments.

The cytosolic phosphatase SHIP-1, SH2-containing inositol-5′-phosphatase, has critical roles in microphages, including generation of alternatively activated macrophages,^39^ regulation of LPS-induced macrophage inflammatory responses^40^ and modulation of myeloproliferation.^11^ Diverse cellular functions have been previously investigated in association of Lyn and SHIP-1,^41,42^ but whether Lyn is involved in SHIP-1 expression or membrane translation^43^ remains unclear. To investigate it, we measured SHIP-1 expression in Lyn-silenced and control MH-S cells at 0, 30 and 60 min post infection of PAO1. Immunoblotting results showed that SHIP-1 expression was induced in control MH-S cells, but excessively induced Lyn-silenced MH-S cells (Figure 3a; Supplementary Figure S2D), indicating that PAO1-dependent SHIP-1 expression in AMs is regulated by Lyn. Further, to elucidate whether Lyn has roles in membrane translocation of SHIP-1, Triton-soluble and Triton-insoluble fractions (lipid rafts) were isolated from Lyn-silenced and control MH-S cells using sucrose density gradients. The fractions were separated by SDS-polyacrylamide gel electrophoresis and examined for the presence of SHIP-1 by immunoblotting. Thirty min post infection, only small portion of SHIP-1 was translocated to the plasma membrane in control MH-S cells, but almost 100% translocated to the plasma membrane in Lyn-silenced MH-S cells (Figure 3b). Confocal laser scanning microscopy also showed an enhanced expression and fast membrane translocation of SHIP-1 in Lyn-silenced MH-S cells, compared with that of control MH-S cells (Figure 3c), again demonstrating that Lyn influences the expression and membrane translocation of SHIP-1. In addition, we successfully blocked its expression by treating Lyn-silenced and control MH-S cells with STAT3 inhibitor (Figure 3d; Supplementary Figure S2E). Immunoblotting showed that when STAT3 expression was inhibited, the expression of SHIP-1 was consistently repressed (Figure 3d; Supplementary Figure S2E), indicating that excessive SHIP-1 expression in Lyn-silenced MH-S cells is caused by Lyn-dependent STAT3 activation.

### SHIP-1 deficiency results in increased survival and reduced inflammatory responses in Lyn^−/−^ mice against PAO1 infection

**Figure 4:**
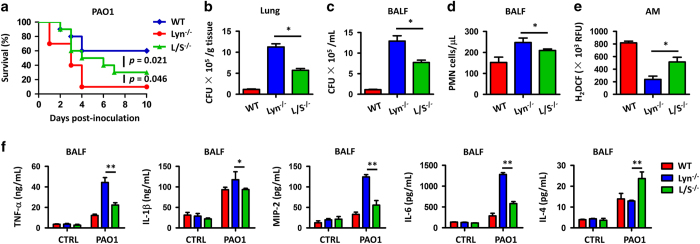
SHIP-1 deficiency contributes to increased survival and reduced inflammatory responses in Lyn^−/−^ mice against PAO1 infection. (**a**) Lyn^−/−^, Lyn^−/−^/SHIP-1^−/−^ (L/S^−/−^) and WT mice were challenged intranasally with 1×10^7^ CFU PAO1 per mouse (10 mice per group) and then observed to 10 days. Kaplan–Meier survival curves were obtained: *P*=0.021 (L/S^−/−^ mice versus WT mice) and *P*=0.046 (L/S^−/−^ mice versus Lyn^−/−^ mice). (**b**) At 24 h post PAO1 infection, bacterial burdens determined in the lungs after homogenization in phosphate-buffered saline. (**c**) Bacterial loads in BALF derived from infected mice. (**d**) PMN percentages versus total nuclear cells in BALF using HEMA-3 staining. (**e**) Superoxide production in AMs detected by an dihydrodichlorofluorescein diacetate assay at a wavelength of 488 nm. (**f**) Inflammatory cytokines in BALF assessed by enzyme-linked immunosorbent assay. Data (mean±s.e.m.) are representative of three independent experiments (one-way analysis of variance with Tukey’s *post hoc*; *P⩽0.05; **P⩽0.01).

To further characterize the critical role of SHIP-1 and Lyn during bacterial infection, we cross-bred Ship-1^−/−^ mice with Lyn^−/−^ mice to generate a double gene knockout mouse (Lyn^−/−^/Ship-1^−/−^, short as L/S^−/−^, Supplementary Figure 1A), and then challenged with PAO1 (10 mice per group). Unexpectedly, although containing a higher mortality compared with WT mice (*P*=0.021, log-rank test), L/S^−/−^ mice had a significantly decreased mortality compared with Lyn^−/−^ mice (*P*=0.046, log-rank test; Figure 4a). We also found that 24 h post PAO1 infection, L/S^−/−^ mice exhibited decreased bacterial burdens in the lungs (Figure 4b), BALF (Figure 4c) and other organs including liver, spleen and kidney, compared with that of Lyn^−/−^ mice (Supplementary Figures 1B–D). PMNs in the BALF of L/S^−/−^ mice were also reduced (Figure 4d), whereas ROS in the AMs of L/S^−/−^ mice were increased (Figure 4e). In addition, the pro-inflammatory cytokines, including TNF-α, MIP-2, IL-1β and IL-6 levels, were significantly reduced in BALF from L/S^−/−^ mice compared with Lyn^−/−^ mice, whereas anti-inflammatory cytokine IL-4 was increased (Figure 4f). These data collectively demonstrated that knockout of SHIP-1 in Lyn^−/−^ mice reversed, at least in part, severe lung damage and inflammatory responses that caused by Lyn deficiency, indicating that SHIP-1 may exert its function in host defense in a Lyn-dependent manner.

### SHIP-1 deficiency leads to reduced NF-κB-dependent transcription in Lyn^−/−^ AMs in PAO1 infection

**Figure 5:**
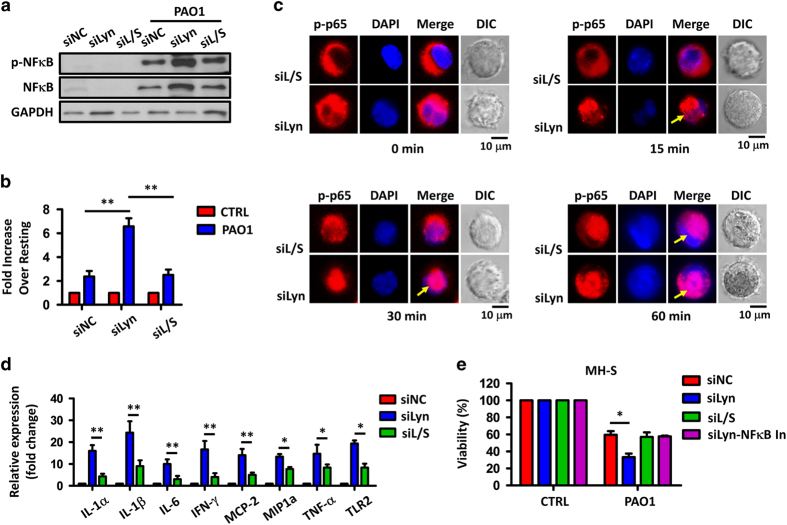
SHIP-1 deficiency results in reduced NF-κB-dependent gene transcription in Lyn^−/−^ AMs response to PAO1 infection. (**a**) MH-S cells were transfected with Lyn siRNA alone or together with SHIP-1 siRNA (and control siRNA) at 5 pM for 48 h. Lyn-silenced, Lyn and SHIP-1 double-silenced, and control MH-S cells were then infected with PAO1 at an MOI of 20:1 for 30 min. Expression and phosphorylation of NF-κB were measured by immunoblotting. (**b**) These cells were then transfected with plasmids encoding the NF-κB-luc (100 ng) for 24 h, and then infected PAO1 at an MOI of 20:1 for 30 min. Cells were lysed and luciferase gene expression was measured. (**c**) These cells were then infected with PAO1 at an MOI of 20:1 for 0, 15, 30 and 60 min. Confocal laser scanning microscopy showed nuclear translocation of p-NF-κB (p-p65) in MH-S cells using immune staining (arrows showing the nuclear translocation). Scale bar, 10 μm. (**d**) Transcripts of TLR2 and indicated cytokines in MH-S cells after PAO1 infection (MOI=20:1, 30 min) quantified by quantitative reverse transcription-PCR. (**e**) Lyn-silenced MH-S cells were treated with NF-κB inhibitor (20 μm) before and during PAO1 infection (MOI=20, 30 min). Lyn-silenced, Lyn and SHIP-1 double-silenced, and control MH-S cells were also infected with PAO1 at an MOI of 20:1 for 30 min. Cell viability of indicated MH-S cells was determined by 3-(4,5-dimethylthiazol-2-yl)-2,5-dimethyltetrazolium bromide assay. Data (mean±s.e.m.) are representative of three independent experiments (one-way analysis of variance with Tukey’s *post hoc*; *P⩽0.05; **P<0.01). In, inhibitor.

PAO1 infection of macrophages induces expression of transcription factor NF-κB, promotes its nucleic translocation and activates its downstream cytokine gene transcription.^44^ It has previously been reported that SHIP-1 positively regulates NF-κB-dependent gene transcription in macrophage RAW264.7 in response to LPS stimulation.^45^ Therefore, we asked whether SHIP-1 and Lyn influenced gene expression driven by NF-κB in AMs in response to PAO1 infection. Immunoblotting results showed that levels of total and phosphorylated NF-κB were increased in the Lyn-silenced MH-S cells, but were inhibited in the Lyn and SHIP-1 double-silenced MH-S cells (Figure 5a; Supplementary Figure S2F). Lyn-silenced, Lyn and SHIP-1 double-silenced, and control MH-S cells were transiently transfected with luciferase reporter plasmids that were dependent on NF-κB binding (NF-κB-luc). NF-κB-dependent transcription of luciferase in these transfected cells were induced 30 min post PAO1 infection (Figure 5b). Compared with that in control MH-S cells, activity of luciferase in Lyn-silenced MH-S cells was significantly higher, but was not changed in Lyn and SHIP-1 double-silenced cells (Figure 5b). Confocal laser scanning microscopy data showed that p-NF-κB was slowly activated and translocated into the nuclei in Lyn and SHIP-1 double-silenced cells, compared with Lyn-silenced MH-S cells (Figure 5c). These data demonstrated that knockdown of SHIP-1 in Lyn-deficient AMs weakened PAO1-induced activation, phosphorylation and nuclear translocation of NF-κB.

We then measured NF-κB-dependent gene transcription in indicated MH-S cells. Quantitative reverse transcription-PCR data showed that 30 min post PAO1 infection, transcriptions of all tested NF-κB-dependent genes, including IL-1α, IL-1β, IL-6, IFN-γ, MCP-2, MIP1α, TNF-α and TLR2, were significantly increased in Lyn-silenced MH-S cells (Figure 5d). Compared with Lyn-silenced MH-S cells, increase of these gene transcription in Lyn and SHIP-1 double-silenced MH-S cells was dampened (Figure 5d), indicating that knockdown of SHIP-1 in Lyn-deficient AMs abolished NF-κB-dependent gene excessive activation. 3-(4,5-Dimethylthiazol-2-yl)-2,5-dimethyltetrazolium bromide assay showed that 30 min post PAO1 infection, cell viability of Lyn-silenced MH-S cells was significantly hampered compared with control cells (Figure 5e). The reduced cell viability was partially restored to the level of control MH-S cells when SHIP-1 was also silenced in Lyn-silenced cells (Figure 5e). In addition, when NF-κB was inhibited, the viability of Lyn-silenced MH-S cells increased to the similar level of Lyn and SHIP-1 double-silenced or control MH-S cells (Figure 5e), indicating a critical role of Lyn, SHIP-1 and NF-κB in AM cell survival during PAO1 infection.

### PAO1-induced MAPK phosphorylation is downregulated in SHIP-1 deficiency AMs

**Figure 6:**
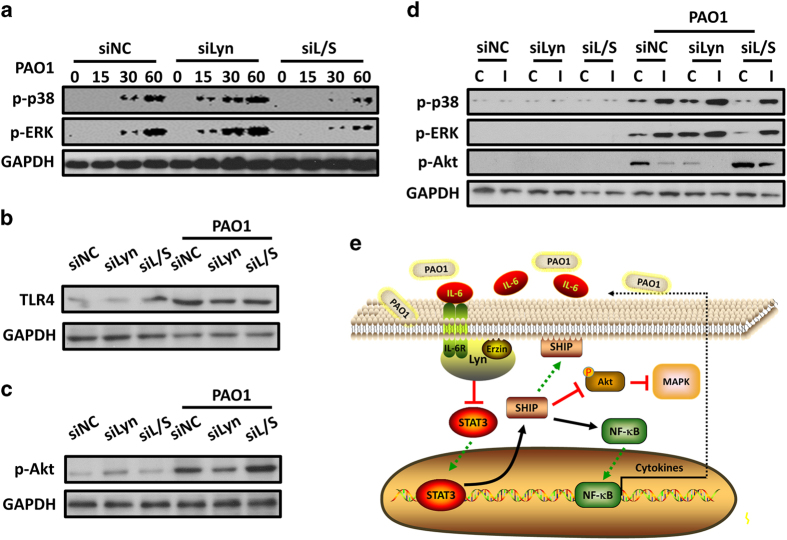
PAO1-induced MAPK phosphorylation is downregulated in SHIP-1 deficiency AMs. (**a**) MH-S cells were also transfected with Lyn siRNA alone or together with SHIP-1 siRNA at 5 pM for 48 h. Lyn-silenced, Lyn and SHIP-1 double-silenced, and control MH-S cells were then infected with PAO1 at an MOI of 20:1 for 0, 15, 30 and 60 min. Phosphorylation of p38 and ERK1/2 was measured by immunoblotting. (**b**, **c**) Expression of TLR4 (**b**) and phosphorylation of Akt (**c**) were measured by immunoblotting in MH-S cells after PAO1 infection (MOI=20:1, 30 min). (**d**) These cells were treated with Akt inhibitor (20 μm) before and during PAO1 infection (MOI=20:1, 30 min). Phosphorylation of Akt, p38 and ERK1/2 was measured by immunoblotting. C, control; I, inhibitor. Data are representative of three independent experiments. (**e**) Schematic representation of Lyn’s role in *P. aeruginosa* infection. Upon PAO1 infection, free Lyn binds IL-6R and Ezrin, to inhibit STAT3 activation and further impairs SHIP-1 activation. Inhibition of SHIP-1 dampens NF-κB activation and its downstream pro-inflammatory cytokines production, as well as reduction MAPK activation by inducing Akt phosphorylation.

MAPK phosphorylation in macrophages is thought to be TLR4-dependent in *P. aeruginosa* infection.^46^ To examine the molecular details of Lyn and SHIP-1 in regulating TLR4 signaling, we next assessed the activation of MAPK in Lyn-silenced, Lyn and SHIP-1 double-silenced, and control MH-S cells. Immunoblotting results indicated that robust phosphorylation of ERK1/2 and p38 was significantly induced in Lyn-silenced MH-S cells, compared with control cells (Figure 6a; Supplementary Figure S2G). In contrast, the enhanced phosphorylation of ERK1/2 and p38 was partially impeded in Lyn and SHIP-1 double-silenced MH-S cells (Figure 6a; Supplementary Figure S2G). To ensure that the signaling differences observed were not due to the differences in the expression of TLR4, we measured TLR4 by immunoblotting and found a similar expression level in all indicated MH-S cells (Figure 6b; Supplementary Figure S2H). These results indicate that Lyn negatively while SHIP-1 positively regulates the activation of ERK and p38, and that this regulation is TLR4-independent.

Previous studies demonstrate that Akt activation leads to a downregulation of LPS-induced MAPK phosphorylation.^40^ To test whether the altered phosphorylation of MAPK is accompanied with different Akt activation, phosphorylation of Akt (Ser473) was compared in Lyn-silenced, Lyn and SHIP-1 double-silenced, and control MH-S cells. The results shown in Figure 6c (and Supplementary Figure S2I) indicate that Akt phosphorylation is induced in control MH-S cells 30 min post PAO1 infection. In contrast, Akt phosphorylation is significantly reduced in Lyn-silenced MH-S cells, but is substantially enhanced in Lyn and SHIP-1 double-silenced MH-S cells after PAO1 infection (Figure 6c; Supplementary Figure S2I). These results suggest that Lyn and SHIP-1 may regulate PAO1-induced MAPK activation by changing Akt phosphorylation. In addition, to test whether induction of Akt in Lyn and SHIP-1 double-silenced MH-S cells would restore PAO1-induced MAPK phosphorylation, MH-S cells were treated with the PI3K inhibitor wortmannin for 1 h before infection of PAO1. The results shown in Figure 6d (and Supplementary Figure S2J) suggest that MAPK phosphorylation was restored in Lyn and SHIP-1 double-silenced MH-S cells of which Akt phosphorylation was suppressed.

## Discussion

In this study, we report a critical regulatory role of Lyn in host defense against *P. aeruginosa* infection, which is exerted in an IL-6/STAT3 signaling axis. We show that Lyn^−/−^ mice have a high susceptibility to *P. aeruginosa* infection manifested with decreased bacterial clearance, heightened pro-inflammatory cytokines and severe lung injury. Built on the situation in *K. pneumoniae* infection as seen in our previous report,^20^ Lyn regulates an IL-6/STAT3 signaling pathway in AMs during *P. aeruginosa* infection by direct binding with IL-6R and cytoskeletal protein Ezrin through its SH2 and SH3 domains. Importantly, our data reveal a mechanism that SHIP-1 was in cooperation with Lyn in regulation of host immune responses. Deficiency in Lyn results in excessive STAT3 activation and thus enhancing SHIP-1 expression and membrane translocation. Lack of SHIP-1 in Lyn^−/−^ mice promotes survival and thereby attenuating inflammatory responses during *P. aeruginosa* infection. Mechanistically, loss of SHIP-1 reduces NF-κB-dependent cytokine production, dampened MAPK activation through a PI3K/Akt pathway (summarized in Figure 6e). Thus, these finding strongly attest that Lyn negatively regulates AM inflammation against *P. aeruginosa* infection through the SHIP-1 and IL-6/STAT3 signaling pathway.

This study again supports a concept that Lyn is critically involved in host inflammatory responses during *P. aeruginosa* infection. Our data showed that disease-related phenotypes in *P. aeruginosa* infected Lyn^−/−^ mice were similar to that in *K. pneumoniae* infected Lyn^−/−^ mice,^20^ including increased mortality, severe lung injury, elevated inflammatory cytokines (IL-6 and TNF-α), increased phosphorylation of p38 and NF-κB. In addition, these indicated phenotypes are also in line with observations in house dust mite-treated Lyn^−/−^ mice,^31^ suggesting that Lyn may render a variety of immune responses to a wide range of microorganisms and other immunogens. On the other hand, bacterial species-dependent roles of Lyn in regulation of immune responses have previously been demonstrated. However, ROS in AMs of Lyn^−/−^ mice were significantly reduced during *P. aeruginosa* infection (Figure 1e), but markedly increased during *K. pneumoniae* infection,^20^ suggesting that Lyn’s influence on ROS production also varies with bacterial species. As Lyn^−/−^ mice were germline knocked out, all these indicated typical disease phenotypes, including decreased bacterial clearance, heightened pro-inflammatory cytokines and severe lung injury in Lyn^−/−^ mice (Figure 1), were on the impact of combined roles of Lyn in various cell types. For example, inhibition of Lyn’s function prevented *P. aeruginosa* internalization in lung epithelial cells,^21^ which may accelerate bacterial dissemination to other organs and caused secondary injury (Supplementary Figure 1). Importantly, Lyn deficiency also significantly inhibited bacterial phagocytosis and timely clearance by AMs,^17,22^ which resulted in the higher bacterial burden in BALF and lung tissue of Lyn^−/−^ mice (Figure 1).

In this study, we focused on the investigation of Lyn’s role in AMs, because the mortality of PAO1-infected mice is highly AM-dependent.^34^ As one of the primary inflammatory cytokine source in the lung and BALF,^35^ excessive activation of AMs increases inflammation and stimulates immune system,^45^ leading to programmed cell death and mice mortality.^45,47^ Previous studies showed that both Lyn and pro-inflammatory cytokine IL-6 were involved in several autoimmune diseases. For example, Lyn-deficient leukocytes, notably B cells, overproduce IL-6, and this establishes an inflammatory environment leading to the activation of B cells and cellular components, thus developing severe autoimmune pathology.^8^ In human multiple myeloma cells, the association of CD45 and Lyn requires the presence of IL-6.^48^ Nevertheless, how Lyn regulates IL-6- and IL-6-dependent signaling pathway is largely unknown. Using co-immunoprecipitation assays, we observed a stable association between IL-6R, Lyn and Ezrin in mouse AMs after PAO1 infection (Figure 2e). Lyn is dominant in this protein complex because in Lyn-deficient AMs, the interactions between IL-6R and Ezrin are diminished (Figure 2e). Using purified peptides containing different Lyn functional domains, we clarified that both of Lyn SH2 and SH3 domains can bind to IL-6R, whereas Lyn kinase domain cannot (Figure 2g). During *P. aeruginosa* infection, an interaction of IL-6 and IL-6R in AMs activates transcript STAT3 to initiate the expression of other inflammatory factors.^36^ Our data showed that interactions between IL-6R, Lyn and Ezrin negatively regulate the IL-6/STAT3 signaling pathway, because respective inhibition of each of these components results in enhanced expression and phosphorylation of STAT3 after PAO1 infection (Figures 2d and i). This indirect regulation of STAT3 by Lyn in AMs is different from that in other cell types. For example, Lyn directly phosphorylates STAT3 to impact proliferation, differentiation or growth arrest in B cells.^49^ In our study, inhibition of Lyn and Ezrin, respectively, resulted in a decreased cell viability after PAO1 infection (Figure 2j), indicating that this complex has a critical role in host defense against *P. aeruginosa*. In addition, we noticed that the IL-6 production was significantly elevated in Lyn-silenced MH-S cells (Figures 2a–c), suggesting that the activation of IL-6/STAT3 pathway may also result from IL-6 stimulation. However, our data showed that expression of IL-6R in Lyn-silenced cells was not changed compared with that of control cells (Figure 2c). We believe that IL-6R expression limits the direct binding of IL-6 and IL-6R in Lyn-silenced MH-S cells, even IL-6 was over produced, indicating that Lyn signaling to IL-6 contributes significantly to the STAT3 activation.

In this study, we identified the mechanism of SHIP-1 along with Lyn in immune responses to *P. aeruginosa* infection. Diverse cellular functions have been previously investigated in association with both Lyn and SHIP-1.^41,42^ SHIP-1 and Lyn are involved in regulation of integrin alpha IIb-β3 signaling in platelets,^41^ and in respect to Ag-triggered degranulation in mast cells.^42^ In macrophages, Lyn and SHIP-1 negatively regulate macrophage colony-stimulating factor-induced Akt activity.^24^ A previous study demonstrated that activation of macrophages by the stimulation of LPS is negatively regulated by a Lyn/PI3K axis and promoted by SHIP-1.^50^ However, the internal relationship between Lyn and SHIP-1 remains unclear. Direct phosphorylation and activation of SHIP-1 by Lyn has been reported with a loss of SHIP-1 activity in Lyn-deficient mast cells.^51^ Our data for the first time showed that the expression and membrane translocation of SHIP-1 is dependent on Lyn in AMs (Figures 3a–c) during *P. aeruginosa* infection, which is an indirect role. Interaction of Lyn and IL-6R inhibits the activation of transcript factor STAT3 (Figure 2d), which further downregulates SHIP-1 expression (Figure 3d). Unlike the STAT3-independent pathway through a SHIP-1/IL-10/TNF-α axis,^52^ our findings indicate a STAT3-dependent SHIP-1 regulation during immune responses. Importantly, we observed that an mitigated disease phenotype in Lyn and SHIP-1 double knockout mice after PAO1 infection, including increased survival, decreased PMN and reduced inflammatory responses, compared with Lyn^−/−^ mice (Figure 4), indicating that SHIP-1 positively regulates host immune responses, which is in line with other studies.^50^

Studies have investigated the molecular mechanisms by which SHIP-1 regulates variable types of inflammation responses, including allergic airway inflammation,^53^ chronic inflammation in the myeloid compartment,^54^ triptolide ameliorates lieocolonic anastomosis inflammation^53^ and LPS-induced inflammatory responses.^50^ Compared with the previous studies, we demonstrate that Lyn collaborating with SHIP-1 acts through both the similar and unique mechanisms to regulate multiple signaling pathways. Our previous and current findings showed that Lyn negatively regulates the activation and nuclear translocation of NF-κB in AMs after both *P. aeruginosa* and *K. pneumoniae* infection. Here we validated that Lyn regulates NF-κB activation and NF-κB-dependent gene transcription through a Lyn/SHIP-1/NF-κB axis (Figure 5). We further demonstrate that Lyn positively regulates bacteria-induced Akt phosphorylation and thus negatively regulates Akt-dependent activation of MAPK in AMs through SHIP-1 (Figure 6). Similar roles for Lyn and SHIP-1 in regulation of Akt were observed in B lymphocytes^55^ and bone marrow-derived macrophages.^40,50^ Studies showed a role for Lyn in controlling MAPK activation is TLR4-dependent.^50,56^ However, our findings showed that expression of TLR4 was not significantly changed in AMs when Lyn was knocked out or Lyn and SHIP-1 were both knocked out (Figure 6), suggesting that Lyn and SHIP-1 also controls MAPK activation in a TLR4-independent way. Despite being more dominant in this infection model for Lyn, SHIP-1 also exhibited a critical role.

In summary, we found that Lyn is required for full resistance to *P. aeruginosa* infection and its deficiency contributes to elevated inflammatory cytokine responses,^57,58^ which resulted in a severe susceptibility to this infection. We for the first time demonstrate that during bacterial infection, a Lyn–IL-6R–Ezrin complex negatively regulates the IL-6/STAT3 signaling pathway in murine AMs. Further, we unraveled that SHIP-1 is under Lyn/STAT3 regulation and has critical roles in host inflammatory responses. Lyn regulates NF-κB activation and NF-κB-dependent gene transcription through a Lyn/SHIP-1/NF-κB axis. In addition, the Lyn/SHIP-1 axis controls MAPK activation in a TLR4-independent manner. Overall, our findings provide deeper insight into the role of Lyn in the regulation of host immune response against *P. aeruginosa* infection and may help identify novel therapeutic approaches to combatting against bacterial infection.
