# Supplementary material for: Lyn prevents aberrant inflammatory responses to Pseudomonas infection in mammalian systems by repressing a SHIP-1-associated signaling cluster
Source: Signal Transduct Target Ther. 2016 Dec 16;1:16032–. doi: 10.1038/sigtrans.2016.32 (PMC5661651; doi:10.1038/sigtrans.2016.32)
Supplement: Supplementary Information [file sigtrans201632-s1.doc]

**Lyn prevents aberrant inflammatory responses to *Pseudomonas* infection in mammalian systems by repressing a SHIP-1-associated signaling cluster**

Li R, et al.


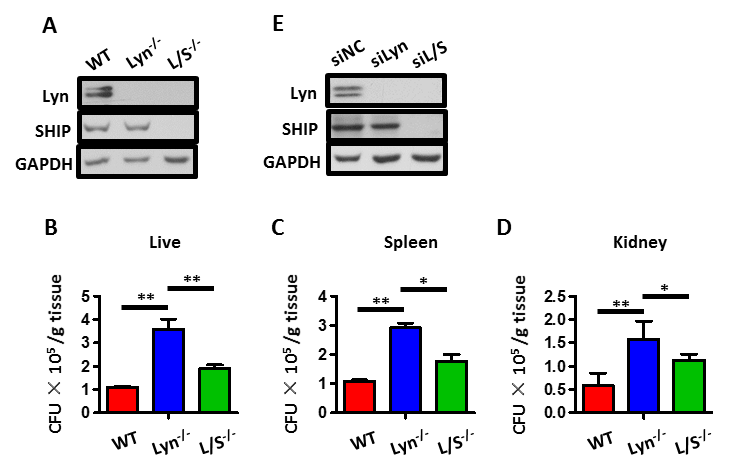


**SI Fig. 1** (**A**) Expression of Lyn and SHIP-1 in lungs of Lyn^-/-^, Lyn^-/-^/Ship-1^-/-^ (L/S^-/-^) and WT mice were detected by immunoblotting. (**B-D**) Bacterial burdens in liver (**B**), spleen (**C**) and kidney (**D**) were significantly increased after 24 h post-PAO1 infection in Lyn^-/-^ mice compared with WT mice, but compared to Lyn^-/-^ mice, bacterial burdens in this organs were significantly decreased. (**E**) MH-S cells were transfected with control siRNA, Lyn siRNA alone or together with SHIP-1 siRNA at 5 pM for 48 h. Expression of Lyn and SHIP-1 of Lyn-silenced, Lyn and SHIP double silenced, and control MH-S cells were detected by immunoblotting. Data are representative of three reproducible experiments expressed as means ± SEM (one-way ANOVA with Tukey’s post hoc; **p* ≤ 0.05; ***p* ≤ 0.005).


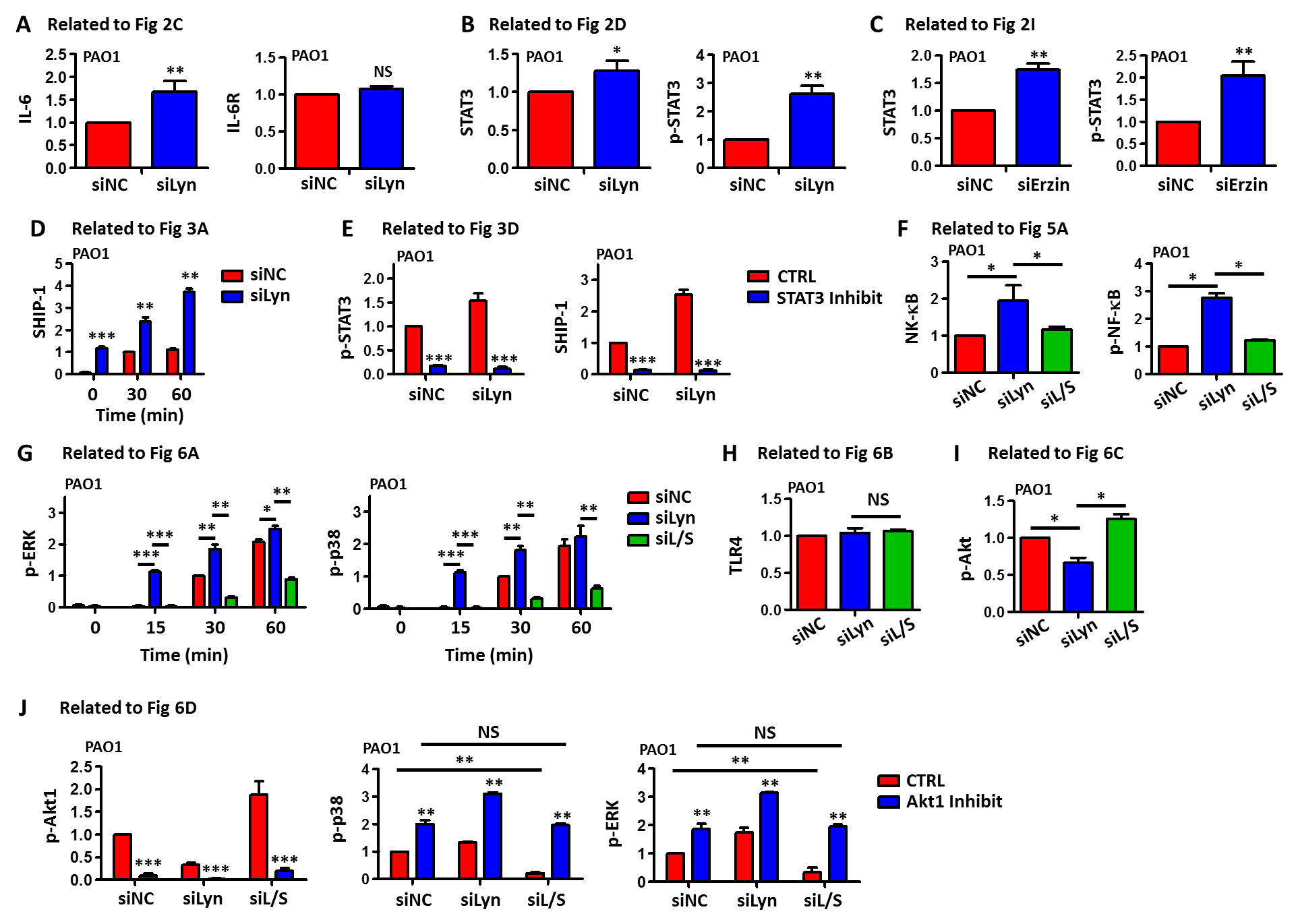


**SI Fig. 2.** Densitometric quantification of the immunoblotting gel data presented in main Figures (in text) using Quantity One software: (**A**) Fig. 2C, (**B**) Fig. 2D, (**C**) Fig. 2I, (**D**) Fig. 3A, (**E**) Fig. 3D, (**F**) Fig. 5A, (**G**) Fig. 6A, (**H**) Fig. 6B, (**I**) Fig. 6C, (**J**) Fig. 6D. Data are representative of three reproducible experiments expressed as means ± SEM (one-way ANOVA with Tukey’s post hoc; **p* ≤ 0.05; ***p* ≤ 0.005; ****p* ≤ 0.001; NS: no significant change).

Table S1. Primers used in this study.

| Name | Sequence |
| --- | --- |
| IL1a F | TGCAGTCCATAACCCATGAT |
| IL1a R | GACAAACTTCTGCCTGACGA |
| IL1b F | TCAGGCAGGCAGTATCACTC |
| IL1b R | CATGAGTCACAGAGGATGGG |
| IFNg F | TCCTTTGGACCCTCTGACTT |
| IFNg R | GTAACAGCCAGAAACAGCCA |
| MCP2 F | GACGCTAGCCTTCACTCCA |
| MCP2 R | GACAGGGACAGCTATGAGCA |
| MIP1a F | TCTCCTACAGCCGGAAGATT |
| MIP1a R | GCCGGTTTCTCTTAGTCAGG |
| TNFa F | GACAGTGACCTGGACTGTGG |
| TNFa R | TGAGACAGAGGCAACCTGAC |
| TLR2 F | GTCAGCTCACCGATGAAGAA |
| TLR2 R | GAGCCCATTGAGGGTACAGT |
| GAPDH F | ACAACTTTGGCATTGTGGAA |
| GAPDH R | GATGCAGGGATGATGTTCTG |
